# Regressive Effect of Myricetin on Hepatic Steatosis in Mice Fed a High-Fat Diet

**DOI:** 10.3390/nu8120799

**Published:** 2016-12-11

**Authors:** Shu-Fang Xia, Guo-Wei Le, Peng Wang, Yu-Yu Qiu, Yu-Yu Jiang, Xue Tang

**Affiliations:** 1 Wuxi School of Medicine, Jiangnan University, Wuxi 214122, China; yuyuqiu1102@aliyun.com (Y.-Y.Q.); doctoryuyu@126.com (Y.-Y.J.); 2 State Key Laboratory of Food Science and Technology, School of Food Science and Technology, Jiangnan University, Wuxi 214122, China; 1601050217@163.com (G.-W.L.); tangxue@jiangnan.edu.cn (X.T.); 3 COFCO Corporation Oilseeds Processing Division, Beijing 100020, China; wpeng@cofco.com

**Keywords:** myricetin, hepatic steatosis, Nrf2, PPARγ, oxidative stress

## Abstract

Myricetin is an effective antioxidant in the treatment of obesity and obesity-related metabolic disorders. The objective of this study was to explore the regressive effect of myricetin on pre-existing hepatic steatosis induced by high-fat diet (HFD). C57BL/6 mice were fed either a standard diet or a HFD for 12 weeks and then half of the mice were treated with myricetin (0.12% in the diet, *w/w*) while on their respective diets for further 12 weeks. Myricetin treatment significantly alleviated HFD-induced steatosis, decreased hepatic lipid accumulation and thiobarbituric acid reactive substance (TBARS) levels, and increased antioxidative enzyme activities, including catalase (CAT), superoxide dismutase (SOD), and glutathione peroxidase (GPx) activities. Microarray analysis of hepatic gene expression profiles showed that myricetin significantly altered the expression profiles of 177 genes which were involved in 12 biological pathways, including the peroxisome proliferator activated receptor (PPAR) signaling pathway and peroxisome. Further research indicated that myricetin elevated hepatic nuclear Nrf2 translocation, increased the protein expression of heme oxygenase-1 (HO-1) and NAD(P)H quinone dehydrogenase 1 (NQO1), reduced the protein expression of PPARγ, and normalized the expressions of genes that were involved in peroxisome and the PPAR signaling pathway. Our data indicated that myricetin might represent an effective therapeutic agent to treat HFD-induced hepatic steatosis via activating the Nrf2 pathway and the PPAR signaling pathway.

## 1. Introduction

Obesity is a condition of energy imbalance that is accompanied by excessive accumulation of lipids in non-adipose tissues [1]. Hepatic steatosis characterized by the accumulation of lipids in the liver is one such process and increasing in prevalence [2]. High-fat diets (HFD), especially those rich in saturated fat and monounsaturated fat could be responsible for the epidemic [3]. Hepatic steatosis and its related inflammatory state (non-alcoholic steatohepatitis, NASH) are the common hepatic complications of obesity and metabolic disorders. HFD-induced dyslipidemia and lipid accumulation initiate the development of hepatic steatosis, and may progress to NASH, fibrosis, cirrhosis and, ultimately, hepatocellular carcinoma [4], which comprises the non-alcoholic fatty liver disease (NAFLD). Excessive triglyceride accumulation in hepatocytes is the hallmark of NAFLD, which is significantly associated with insulin resistance in liver [5]. Based on the results of animal studies and epidemiological investigations, a two-hit hypothesis has been proposed for the pathogenesis of NAFLD: the first hit is excessive fat accumulation in the liver, and the second hit is oxidative stress (OS) that initiates hepatic steatosis to develop into NASH [6]. Although hepatic steatosis is often self-limited, it is necessary to treat it to avoid its progression to more serious diseases. Currently, no treatments have been established for NAFLD beyond management of comorbidities and weight loss [4]. Lifestyle intervention and pharmacotherapy to treat hepatic steatosis are limited because of poor compliance and side effects. As a result, new approaches to improve hepatic steatosis are urgently necessary.

Rodent research and cell culture experiments demonstrated that antioxidant supplementation could effectively improve hepatic steatosis through attenuating oxidative stress and regulating signaling molecules [7]. Green tea extract attenuated hepatic stetosis by inhibiting adipose lipogenesis, restoring hepatic antioxidant defenses, as well as decreasing hepatic lipid peroxidation and inflammatory responses in *ob/ob* mice [8]. Niacin has also been demonstrated to effectively prevent and reverse experimental hepatic steatosis through decreasing hepatic triglyceride synthesis and lipid peroxidation [9]. When obese mice were treated with an NADPH oxidase inhibitor, reactive oxygen species (ROS) production in adipose tissue was decreased, and diabetes, hyperlipidemia, and hepatic steatosis were improved [10]. Thus, a need exists to verify approaches that alleviate the development and progression of hepatic steatosis and oxidative stress.

Myricetin, (3,5,7,3′,4′,5′-hexahydroxyflavone), a naturally occurring flavonoid, is widely distributed in fruits, vegetables, tea, and medicinal herbs and has been demonstrated to exert many bioactivities, including antioxidant, anti-inflammation, anti-tumor, neuroprotective and cardioprotective properties [11,12]. Myricetin reduced oxidative stress, inhibited hyperglycemia and glucose uptake, decreased hepatic triglyceride and cholesterol contents, and ameliorated liver injury [12,13,14]. Since initial lipid deposition in liver, and subsequent oxidative stress, is involved in NAFLD, myricetin may mitigate the “multiple hits” of NAFLD due to its hypolipidemic and antioxidant actions. The present study was designed to better define the regressive effect of myricetin on pre-existing hepatic steatosis induced by HFD.

## 2. Materials and Methods

### 2.1. Animals

C57BL/6 male mice (38, four-week old) were obtained from Model Animal Research Center of Nanjing University (Nanjing, Jiangsu, China) and housed in a controlled environment (a 12 h/12 h light/dark cycle, 08:00 h to 20:00 h, humidity: 60% ± 5%, temperature: 23 ± 2 °C). After acclimatization for one week on standard laboratory chow, the mice were randomly divided into a control group (Con, 16 mice fed a standard diet of 10% energy from fat) and a HFD group (22 mice fed a HFD diet of 45% energy from fat). The diets were based on a modification of the recommendations of American Institute of Nutrition Rodent Diets (AIN-93). After 12 weeks of feeding, six mice were randomly selected from the HFD group and sacrificed. The liver was harvested and Oil Red O staining was conducted to verify whether the hepatic steatosis was developed. The results showed that five mice, which were about 83% of the total mice, suffered from hepatic steatosis, indicating that the animal model of hepatic steatosis was successfully established. Then eight mice were randomly selected from each group and fed their respective diets with additional 0.12% myricetin (≥98% by high performance liquid chromatography, Aladdin Reagent Co., Shanghai, China) according to the previously published literature [13]. Thus, the present study included four groups: (i) Con; (ii) control diet with 0.12% myricetin (CM); (iii) HFD; and (iv) high-fat diet with 0.12% myricetin (HM). Feeding of all mice with their respective diets (two mice per cage) continued fora further 12 weeks. The animals had free access to the test diets and purified water. All mice were weighed weekly, and food intake was also recorded. All of the experimental procedures were approved by the Jiangnan University Institutional Animal Use and Care Committee (JN No. 5 2015) and according to the National Institutes of Health Guide for Care and Use of Laboratory Animals.

After the feeding period, all mice were fasted overnight and slightly anesthetized with pentobarbital. Blood from the orbital sinus was collected into anticoagulant tubes and plasma was separated after centrifugation (2500× *g* for 15 min at 4 °C) and stored at −20 °C until analyses. Livers were harvested and weighed. Next, fat compartments that included perirenal, epididymal, and mesenteric fat were thoroughly removed and weighed. All the tissues were snap-frozen with liquid nitrogen and stored at −80 °C. Portions of liver were collected into RNALater (Ambion Inc., Austin, TX, USA) for real-time quantitative PCR analysis. The experiments were conducted between 8:00 and 10:00 to minimize possible circadian mRNA expression variation.

### 2.2. Indirect Calorimetric Analysis

The comprehensive laboratory animal monitoring system (CLAMS; Columbus Instruments, Inc., Columbus, OH, USA) was used to evaluate respiratory exchange ratio (RER), energy expenditure (EE = (3.815 + 1.232 × RER) × VO_2_), and ambulatory activity. One week before the final sacrifice, each mouse was placed in the CLAMS for 24 h for measurement of all in vivo parameters, which include oxygen consumption, carbon dioxide production, and RER. Ambulatory activity was monitored in both horizontal and vertical directions using infrared beams to count the beam breaks during the experiment. Each time the mice were allowed to acclimatize in individual metabolic cages for one day and then the data of the second day were used for further analysis.

### 2.3. Plasma Biochemical Analysis

Fasting blood glucose (FBG) was assayed with a glucometer (One Touch; LifeScan Inc., Milpitas, CA, USA). Plasma insulin concentrations were analyzed by specific ELISA kits (Mercodia, Uppsala, Sweden). Homeostatic model assessment index of insulin resistance (HOMA-IR) was calculated as ((insulin, μUI/mL) plasma × (glucose, mmol/L) plasma)/22.5. Plasma total cholesterol (TC), low-density lipoprotein cholesterol (LDL-C), high-density lipoprotein cholesterol (HDL-C), and triglyceride (TG) concentrations, as well as aspartate and alanine aminotransferase (AST and ALT) activities were determined by the corresponding enzymatic colorimetric assay kits (Nanjing Jiancheng Bioengineering Institute, Nanjing, Jiangsu, China) according to the manufacturer’s instructions. Plasma TG levels were determined by the glycerol phosphatase oxidase–phenol4-amino antipyrene peroxidase (GPO-PAP) method, and TC levels were determined by the cholesterol oxidase–phenol4-amino antipyrene peroxidase (CHOD-PAP) method. Plasma HDL-C and LDL-C levels were assayed by standardized selective precipitation methods, using phosphotungstic acid/MgCl_2_ and polyvinyl sulfate as precipitating reagents, respectively.

### 2.4. Hepatic Oxidative Stress Biomarker Determination

Thiobarbituric acid reactive substances (TBARS) levels, catalase (CAT), glutathione peroxidase (GPx), superoxide dismutase (SOD) activities, and protein contents in liver were all determined by corresponding kits obtained from Nanjing Jiancheng Bioengineering Institute (Nanjing, Jiangsu, China) according to the instructions of the manufacturer. TBARS level was measured by monitoring the absorbance at 532 nm using 1,1,3,3-tetramethoxypropane as the standard. CAT was determined colorimetrically at 620 nm and expressed as 1 mol of H_2_O_2_ consumed/min. SOD activity was determined based on its ability to inhibit the reduction of nitrazobluetetrazolium (NBT). A unit of enzyme activity was expressed as 50% inhibition of NBT reduction/min. GPx activity was measured through the glutathione (GSH)/nicotinamide adenine dinucleotide phosphate (NADPH)/glutathione reductase (GR) system. H_2_O_2_ was used as the substrate. Hepatically reduced glutathione (GSH) levels were determined by a fluorometric method with the use of *o*-phthalaldehyde (OPT) as a fluorescent reagent [15]. Protein contents were determined by bicinchoninic acid (BCA) methods using a BCA commercial kit (Beyotime Institute of Biotechnology, Nantong, Jiangsu, China).

### 2.5. Liver Histology

Liver samples (*n* = 4) from the same position were randomly selected from each group and immersed in 4% paraformaldehyde and paraffin embedded sections were stained with hematoxylin and eosin (H & E, Baso, Taipei, Taiwan). Oil Red O (Baso, Taipei, Taiwan) staining for liver samples (*n* = 4) was also conducted after embedded in OCT compound (Sakura Finetech, Tokyo, Japan). All of the pathological sections were observed under a light microscope (Leica DM4000B, Leica, Wetzlar, Germany).

### 2.6. Hepatic Lipid Content Determination

Hepatic lipids were measured using commercial kits (Wako Pure Chemical Industries, Osaka, Japan) according to the manufacturer’s instructions. Briefly, the liver samples were extracted with mixed solvents of methanol-/chloroform (*v/v* = 1:2), followed by centrifugation, and the supernatants were used for further analysis. Protein contents in the supernatants were analyzed by BCA methods.

### 2.7. Nimblegen Gene Chip Microarray

In order to find the possible mechanism for ameliorative effects of myricetin on HFD-induced hepatic steatosis, microarray analysis was used to have a wide understanding of the altered genes and pathways that might be involved. Nimblegen gene chip microarray analysis was performed at CapitalBio Corporation (Beijing, China). Samples from HFD and HM groups were isolated from the frozen livers (*n* = 3 for each group) using Trizol reagent (Invitrogen, Carlsbad, CA, USA) and was further purified using NucleoSpin^®^ RNA clean-up (Macherey-Nagel, Duren, Germany). Array hybridization, washing, and scanning were conducted according to the Nimblegen’s Expression user’s guide. In a comparison analysis, two-class unpaired method in the Significant Analysis of Microarrays (SAM, version 3.02, Stanford University, Stanford, CA, USA) was performed to identify significantly differentially expressed genes (DEGs) between HFD and HM groups. The DEGs were selected and put into Pathway-Express in Onto-Tools [16]. Pathway-Express searches the Kyoto Encyclopedia of Genes and Genomes (KEGG) pathway database for each input gene, and the impact analysis was performed in order to build a list of all associated pathways [17]. An impact factor (IF) is calculated for each pathway incorporating parameters, such as the normalized fold change of the DEGs, the statistical significance of the set of the pathway genes, and the topology of the signaling pathway. The corrected gamma *p*-value is the *p*-value provided by the impact analysis. The differences were considered to be significant when the corrected gamma *p*-value was less than 0.05.

### 2.8. Real-Time Quantitative RT-PCR Analyses

Total RNA was isolated from frozen livers using Trizol (Invitrogen, Carlsbad, CA, USA), and reverse transcribed to cDNA according to the manufacturer’s instructions (Promega, Madison, WI, USA). Platinum Taq polymerase (Life Technologies, Gaithersburg, MD, USA) and SYBR Green I dye (SYBR Green Master Mix, Bioneer, Taejon, Korea) was used to measure in the exponential phase of amplification by an ABI prism 7500 Sequence Detection System (Applied Biosystems, Foster City, CA, USA). Samples were run in triplicate for both the genes of interest and β-actin. The primers for the genes were provided by Shenggong Biotechnology (Shanghai, China). The gene expression was normalized to β-actin. Melting curve analysis was applied to evaluate the specificity of the amplified PCR products.

### 2.9. Western Blotting

In order to determine the hepatic protein expression of PPARγ, NQO1, and HO-1, total protein was isolated from the liver in a cold radio-immunoprecipitation assay (RIPA) lysis buffer (Beyotime Institute of Biotechnology, Nantong, Jiangsu, China) with 1% phosphatase inhibitor cocktail and 1% phenylmethanesulfonyl fluoride (PMSF). To determine the nuclear translocation of Nrf2, the supernatants from the first step were gathered and re-centrifuged. The pellet was re-suspended in buffer to extract the hepatic nuclear protein. After the protein was extracted, equal protein contents were transferred to polyvinylidene fluoride (PVDF) membranes (Millipore, Billerica, MA, USA). The membranes were blocked in Tris-buffered saline (TBS) containing 5% (*w/v*) BSA and thereafter incubated with the primary antibodies, including Nrf2 (Santa Cruz Biotechnology, Santa Cruz, CA, USA), NQO1 (Santa Cruz Biotechnology, Santa Cruz, CA, USA), HO-1 (Santa Cruz Biotechnology, Santa Cruz, CA, USA), PPARγ (Santa Cruz Biotechnology, Santa Cruz, CA, USA), GAPDH (Santa Cruz Biotechnology, Santa Cruz, CA, USA), and Lamin B1 (Serotec Ltd., Oxford, UK) at 4 °C overnight. Then, the blotted membrane was incubated with the secondary antibody (anti-rabbit peroxidase conjugate, 1:5000 dilutions in Tris-buffered saline containing 0.1% Triton X-100 (TBST); Cell Signaling Technology, Beverly, MA, USA) for 1 h at room temperature. Bands were visualized by enhanced chemiluminescence using an enhanced chemiluminescence (ECL) Western Blotting Detection kit (Amersham Biosciences, Piscataway, NJ, USA) using a Bio-Rad ChemiDocTM XRS system (Bio-Rad Laboratories, Inc., Hercules, CA, USA). The protein quantity was determined by densitometry analysis using ImageJ software (version 1.47, National Institutes of Health, Bethesda, MD, USA).

### 2.10. Statistical Analysis

Data were expressed as mean ± SEM. Between group differences of microarray data were performed by univariate analysis using Student’s *t-*test. All other data were analyzed using one-way ANOVA with post-hoc Duncan’s test. Statistical significance was determined as *p* < 0.05. Analysis was done with SPSS 17 (SPSS, Inc., Chicago, IL, USA).

## 3. Results

### 3.1. Effects of Myricetin on Body Weight, Food Intake, and Tissue Weight in HFD-Fed Mice

**Figure 1:**
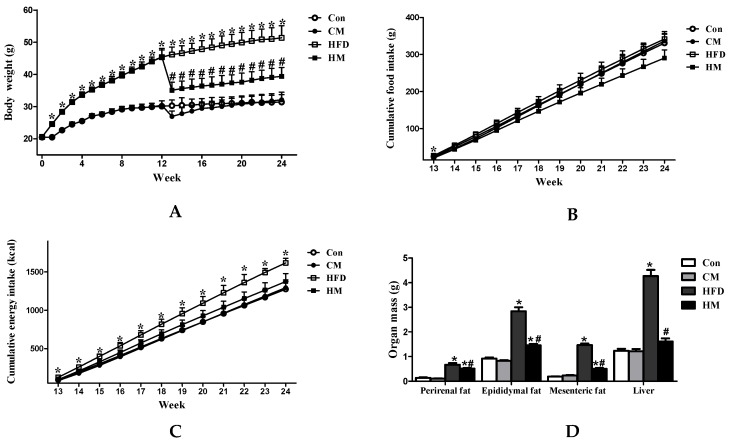
Effects of myricetin on body weight, cumulative food intake and energy intake, and organ mass of mice fed with a high-fat diet. (**A**) Body weight; (**B**) cumulative food intake; (**C**) cumulative energy intake; and (**D**) perirenal, epididymal, and mesenteric fat and liver weight. Values were presented as mean ± SEM (*n* = 8). Con, control diet; CM, control diet with additional 0.12% myricetin; HFD, high-fat diet; HM, high-fat diet with additional 0.12% myricetin. * *p* < 0.05 vs. the Con group; ^#^ *p* < 0.05 vs. the HFD group.

As illustrated in Figure 1, following dietary treatment for 12 weeks, mice in the HFD group exhibited significantly higher body weight than mice in control diet (Figure 1A, *F*_(3, 28)_ = 18.22, *p* < 0.0001). When half mice were administered with 0.12% myricetin, their body weight showed a sharp decrease in the 13th week and began to gradually increase in the later 11 weeks. Finally, HM mice had significantly lower body weight than HFD mice (*F*_(3, 28)_ = 10.52, *p* < 0.0001). The cumulative food intake after grouping demonstrated that HFD mice had significantly higher food intake than Con mice only in the 13th week. No significance was observed on the cumulative food intake among the four groups in the end of the experiment (*F*_(3, 12)_ = 1.13, *p* = 0.377). However, because of the different energy densities between control diet and high-fat diet, HFD mice showed significantly cumulative energy intake compared to Con mice in the later 12 weeks, and HM mice had decreased energy intake, but the difference was not significant (Figure 1B, *F*_(3, 12)_ = 2.97, *p* = 0.075).

Analysis of different fat compartments revealed that perirenal (*F*_(3, 28)_ = 49.90, *p* < 0.0001), epididymal (*F*_(3, 28)_ = 105.73, *p* < 0.0001) and mesenteric fat pad masses (*F*_(3, 28)_ = 246.17, *p* < 0.0001) of HFD mice were significantly higher than those of Con mice. Myricetin treatment significantly reduced the white adipose tissue accumulation compared to HFD mice, but failed to normalize these indexes relative to Con mice. HFD mice showed remarkably increased liver weight compared to Con mice (*F*_(3, 28)_ = 93.13, *p* < 0.0001), which could be normalized by myricetin treatment (Figure 1C). Myricetin, per se, did not affect body weight, energy intake, or tissue weight in mice.

### 3.2. Effects of Myricetin on FBG and Plasma Parameters in HFD-Fed Mice

As illustrated in Table 1, after 24 weeks, HFD mice demonstrated significantly increased FBG (*F*_(3, 28)_ = 3.43, *p* = 0.031), insulin (*F*_(3, 28)_ = 3.12, *p* = 0.042), and HOMA-IR levels compared to Con mice, which could be alleviated by myricetin treatment. In addition, plasma TG (*F*_(3, 28)_ = 6.26, *p* = 0.002), TC (*F*_(3, 28)_ = 14.40, *p* < 0.0001), and LDL-C (*F*_(3, 28)_ = 8.88, *p* < 0.0001) levels were significantly increased and HDL-C levels were significantly decreased in HFD group compared to Con group. Myricetin treatment significantly improved TC, TG, LDL-C, and HDL-C levels. Myricetin, per se, did not affect FBG and plasma parameters in mice.

### 3.3. Effects of Myricetin on RER, Energy Expenditure and Ambulatory Activities in HFD-Fed Mice

**Figure 2:**
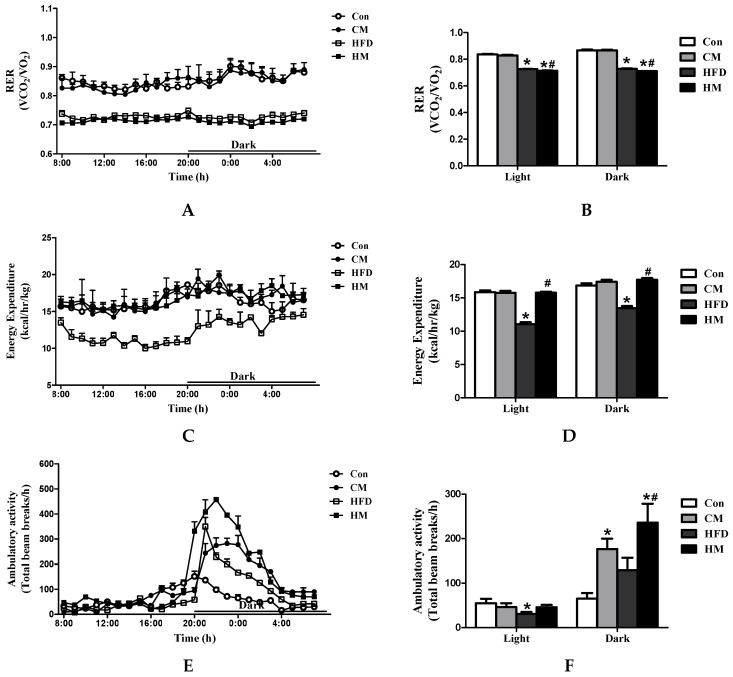
Myricetin increased energy expenditure and promoted the utilization of lipids as fuels. (**A**,**B**) RER; (**C**,**D**) energy expenditure; and (**E**,**F**) ambulatory activities of mice that were fed myricetin-enriched diets for additional 12 weeks after 12 weeks of Con or HFD feeding. Bar graphs represent mean values during the light and dark cycles. Values were presented as mean ± SEM (*n* = 8). Con, control diet; CM, control diet with additional 0.12% myricetin; HFD, high-fat diet; HM, high-fat diet with additional 0.12% myricetin. * *p* < 0.05 vs. the Con group; ^#^ *p* < 0.05 vs. the HFD group.

Myricetin successfully reduced body weight and fat pad masses compared to HFD mice. Apart from the difference in energy intake, we hypothesized that myricetin might exert these effects through increasing energy expenditure. Thus, we used indirect calorimetry to prove the hypothesis. Determinations of RER over a 24 h period demonstrated that HM mice showed decreased RER values than HFD mice in the daytime (*F*_(3, 44)_ = 354.14, *p* < 0.0001) and nighttime (*F*_(3, 44)_ = 349.48, *p* < 0.0001) (Figure 2A,B), indicating a shift in metabolism toward an increase in the utilization of lipids as substrate in mice that receiving myricetin treatment. Additionally, HM mice had significantly higher energy expenditure than HFD mice throughout the whole day (Figure 2C,D). Although no significance on ambulatory activity was observed between HFD and HM mice during the daytime (*F*_(3, 44)_ = 1.71, *p* = 0.179), the difference reached statistically significant levels during the dark period (*F*_(3, 44)_ = 6.55, *p* = 0.001, Figure 2E,F). Myricetin, per se, had no effects on RER or energy expenditure, but remarkably increased ambulatory activities during the nighttime.

### 3.4. Effects of Myricetin on Hepatic Steatosis and Liver Function in HFD-Fed Mice

**Figure 3:**
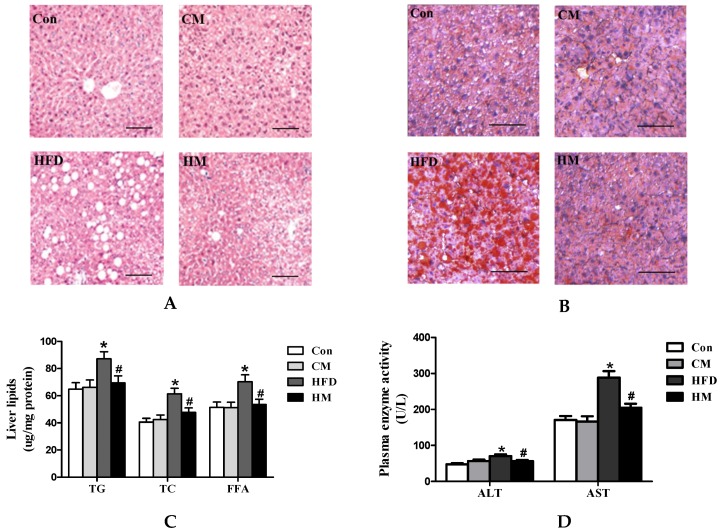
Myricetin reduced hepatic lipid accumulation and increased liver function of mice fed with a high-fat diet. (**A**) Representative images of liver H & E staining (*n* = 4); (**B**) representative images of liver Oil Red O staining (*n* = 4); (**C**) hepatic TG, TC, FFA levels (*n* = 8); (**D**) plasma ALT and AST activities (*n* = 8). Values were presented as mean ± SEM. Scale bars indicate 50 μm. Con, control diet; CM, control diet with additional 0.12% myricetin; HFD, high-fat diet; HM, high-fat diet with additional 0.12% myricetin. * *p* < 0.05 vs. the Con group; ^#^ *p* < 0.05 vs. the HFD group.

HFD mice demonstrated prominent and significant formation of lipid vacuoles in hepatocytes compared with Con mice, while such alterations were relieved by myricetin treatment (Figure 3A). Oil Red O staining of liver sections also confirmed that myricetin significantly reduced HFD-induced hepatic lipid accumulation (Figure 3B). Compared to Con mice, HFD mice had greater hepatic total lipids due to augmentation in TG (*F*_(3, 28)_ = 3.95, *p* = 0.018), TC (*F*_(3, 28)_ = 7.50, *p* = 0.001), and FFA (*F*_(3, 28)_ = 4.51, *p* = 0.011) concentrations, which were fully normalized by myricetin (Figure 3C). Plasma ALT (*F*_(3, 28)_ = 5.99, *p* = 0.003) and AST (*F*_(3, 28)_ = 16.56, *p* < 0.0001) activities were also significantly decreased by myricetin administration (Figure 3D). Taken together, these results suggested that myricetin played a positive role in the alleviation of HFD-induced hepatic steatosis. Myricetin, per se, had no effects on liver function and histological appearance.

### 3.5. Effects of Myricetin on Hepatic Biological Pathways in HFD-Fed Mice

In microarray analysis 177 genes in the HM group showed more than two-fold higher or lower expression levels compared with those in the HFD group. These 177 genes were put into Pathway-Express, searched the KEGG pathways in the Onto-Tools database for each input gene, and built a list of pathways. Herein the biological pathways more than three DEGs were considered to be significantly changed. KEGG annotation showed that myricetin affected 12 biological pathways and the top 10 significantly affected pathways were shown in Table 2, in which the PPAR signaling pathway was one of the most significantly affected pathways since it had the highest impact factor and six DEGs (*Cd36*, *Scd1*, *Cyp7a1*, *Lpl*, *Pparγ*, and *Pck1*). Furthermore, peroxisome was also significantly changed by HM, in which antioxidative genes, such as *Sod2*, *Prdx1*, and *Prdx5* were all significantly down-regulated.

Pathway-Express was used for the pathway impact analysis in order to build a list of all associated pathways. An impact factor (IF) is calculated for each pathway incorporating parameters such as the normalized fold change of the differentially expressed genes, the statistical significance of the set of pathway genes and the topology of the signaling pathway. The corrected gamma *p*-value was provided by the impact analysis. The top 10 pathways that were significant at the 5% level on corrected *p*-values were presented. Altered genes were as follows: *Cd36* (–2.06), *Scd-1* (–2.49), *Cyp7a1* (2.70), *Lpl* (–2.45), *Pparg* (–2.10), *Pck1* (2.05), *Acot1* (2.43), *Acot3* (2.36), *Sod2* (2.32), *Prdx5* (2.28), *Prdx1* (2.08), *C4b* (–3.59), *Plg* (–6.35), *Serpina1a* (4.22), *Arnt* (–3.32), *Jun* (–2.15), *Raf1* (–4.16), *Araf* (2.09), *pla2g2d* (3.12), *Plcb1* (2.35), *Pla2g6* (–2.07), *Cxcl10* (–2.57), *Spp1* (3.00), *Srebf1* (–2.22), and *Ntrk2* (–2.11). Numbers in the parentheses indicated the ratio changes. Positive numbers indicated up-regulation of the HM group relative to the HFD group. Negative numbers indicated down-regulation of the HM group relative to the HFD group.

### 3.6. Effects of Myricetin on Expressions of PPAR Signaling Pathway-Related Genes and PPARγ Protein Expression

**Figure 4:**
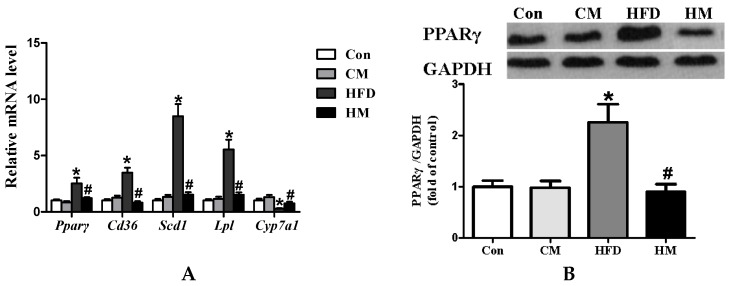
Myricetin normalized expressions of genes involved in lipid metabolism (**A**) and decreased protein expression of hepatic PPARγ (**B**). Values were presented as mean ± SEM (*n* = 8). Con, control diet; CM, control diet with additional 0.12% myricetin; HFD, high-fat diet; HM, high-fat diet with additional 0.12% myricetin. * *p* < 0.05 vs. the Con group; ^#^ *p* < 0.05 vs. the HFD group.

Previous research demonstrated that the PPAR signaling pathway was related to lipid metabolism [18]. According to the present microarray analysis results, the PPAR signaling pathway was the most significantly affected, indicating that the biological pathway might be a target for beneficial effects of myricetin on hepatic steatosis. The DEGs that were involved in the pathway and were important in lipid metabolism (including *Pparγ*, *Cd36*, *Scd1*, *Lpl*, and *Cyp7a1*) were further determined by qPCR. HFD induced significant down-regulation of *Cyp7a1* (*F*_(3, 28)_ = 8.30, *p* < 0.0001), along with remarkable up-regulation of *Pparγ* (*F*_(3, 28)_ = 7.63, *p* = 0.001), *Cd36* (*F*_(3, 28)_ = 22.82, *p* < 0.0001), *Scd1* (*F*_(3, 28)_ = 40.96, *p* < 0.0001), and *Lpl* (*F*_(3, 28)_ = 21.26, *p* < 0.0001), which could be totally normalized by myricetin treatment (Figure 4A). Considering the fact that PPARγ in the liver is related to the regulation of glucose and lipid metabolism by targeting on its responsive genes, such as *Lpl* and *Cd36*, Western blotting analysis was further used to determine the protein expression of PPARγ. As shown in Figure 4B, HFD consumption induced significantly increased protein expression of PPARγ in the liver (*F*_(3, 28)_ = 9.65, *p* < 0.0001), which could be attenuated by myricetin treatment. These data demonstrated that myricetin treatment ameliorated the HFD-induced hepatic steatosis, which might be associated with the PPAR signaling pathway. Myricetin, per se, had no effects on expressions of genes involved in lipid homeostasis and hepatic PPARγ protein expression.

### 3.7. Effects of Myricetin on Hepatic Redox Status in HFD-Fed Mice

**Figure 5:**
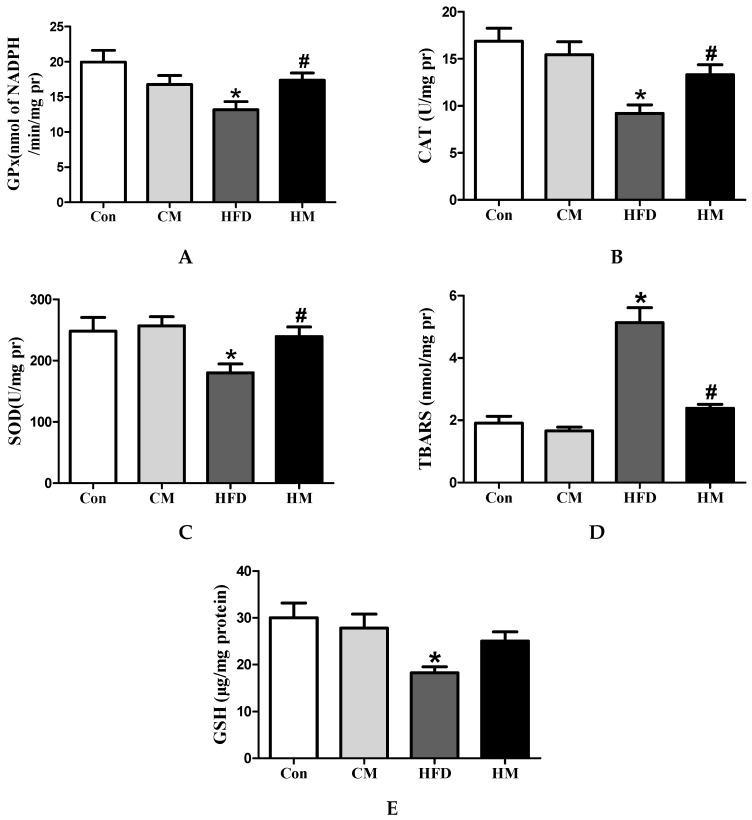
Myricetin alleviated hepatic oxidative stress induced by a high-fat diet. (**A**) GPx; (**B**) CAT; (**C**) SOD; (**D**) TBARS; and (**E**) GSH levels. Values were presented as mean ± SEM (*n* = 8). Con, control diet; CM, control diet with additional 0.12% myricetin; HFD, high-fat diet; HM, high-fat diet with additional 0.12% myricetin. * *p* < 0.05 vs. the Con group; ^#^ *p* < 0.05 vs. the HFD group.

To evaluate the role of myricetin on oxidative stress, hepatic redox status of related biomarkers were determined. As illustrated in Figure 5, HFD consumption caused serious oxidative stress in the liver, as evidenced by significantly reduced GPx (*F*_(3, 28)_ = 4.53, *p* = 0.01), SOD (*F*_(3, 28)_ = 4.05, *p* = 0.017), CAT (*F*_(3, 28)_ = 7.76, *p* = 0.001) activities, and GSH (*F*_(3, 28)_ = 4.23, *p* = 0.014) levels, along with increased TBARS (*F*_(3, 28)_ = 33.18, *p* < 0.0001) levels in HFD group. Myricetin treatment fully normalized GPx, CAT, and SOD activities and lowered TBARS levels, but failed to significantly increase the GSH levels. These results demonstrated remarkable antioxidative characteristics of myricetin. Myricetin, per se, had no effects on hepatic redox status.

### 3.8. Effects of Myricetin on Expressions of Oxidative Stress-Related Genes and Nrf2 Pathway

**Figure 6:**
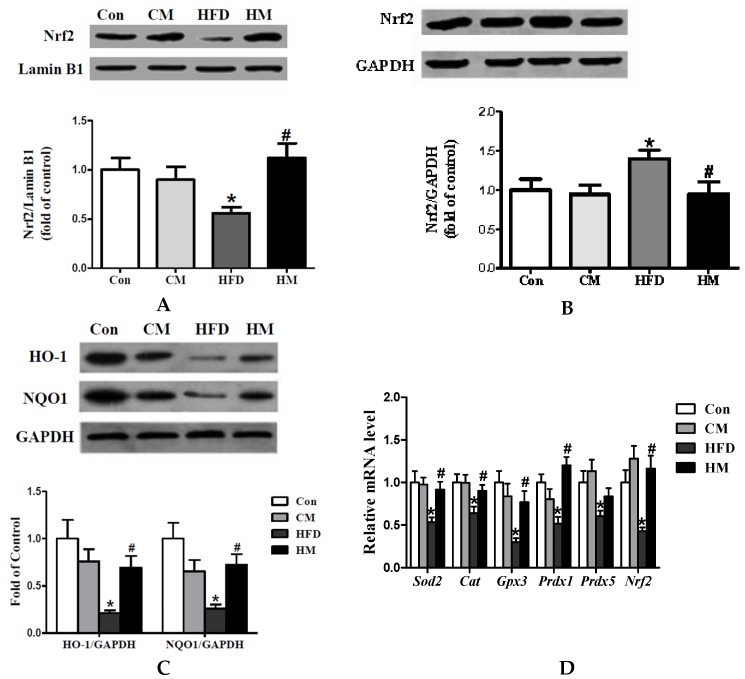
Myricetin activated the hepatic Nrf2 pathway and normalized expressions of genes involved in oxidative stress. (**A**) Protein expression of hepatic nuclear Nrf2; (**B**) protein expression of hepatic cytosolic Nrf2; (**C**) protein expression of hepatic NQO1 and HO-1; and (**D**) relative expression of genes involved in oxidative stress. Values were presented as mean ± SEM (*n* = 8). Con, control diet; CM, control diet with additional 0.12% myricetin; HFD, high-fat diet; HM, high-fat diet with additional 0.12% myricetin. * *p* < 0.05 vs. the Con group; ^#^ *p* < 0.05 vs. the HFD group.

To explore whether the alleviation of myricetin on hepatic steatosis was related to the activation of Nrf2 pathway, we measured the protein expression of nuclear and cytosolic Nrf2, as well as NQO1 and HO-1. The results showed that protein expression of hepatic nuclear Nrf2 was significantly lower (*F*_(3, 28)_ = 4.10, *p* = 0.016) and cytosolic Nrf2 was evidently higher (*F*_(3, 28)_ = 2.80, *p* = 0.058) in HFD group than in control mice, which was mitigated by myricetin treatment (Figure 6A,B). Protein expression of NQO1 (*F*_(3, 28)_ = 6.42, *p* = 0.002) and HO-1 (*F*_(3, 28)_ = 6.04, *p* = 0.003), the targets of Nrf2, was also decreased by HFD and normalized by myrcetin treatment (Figure 6C). Real-time quantitative PCR data showed that the expressions of DEGs (*Sod2*, *Prdx1*, and *Prdx5*) selected from peroxisome pathway, along with oxidative stress related *Cat*, *Gpx3*, and *Nrf2*, were noticeably down-regulated by HFD consumption and normalized by co-administration of myricetin, except for *Prdx5* (Figure 6D). These results indicated that myricetin could increase hepatic nuclear Nrf2 translocation to elevate antioxidative capacity possibly via activating Nrf2 pathway. Myricetin, per se, had no effects on the expressions of genes involved in oxidative stress and the Nrf2 pathway.

## 4. Discussion

NAFLD, as an emerging health problem worldwide, has an estimated prevalence of 20%–40% in Western countries [19]. Epidemiological surveys have also revealed that community prevalence of NAFLD was about 15% in eastern and southern areas of China [20]. Although the pathogenesis of NAFLD is not entirely understood, the “two hits” hypothesis is widely accepted [6]. Hepatic steatosis, the “first hit” of NAFLD, is characterized by fat infiltration and excessive lipid accumulation in the liver, accompanied by an elevated liver/body ratio and higher plasma levels of enzyme markers of liver damage. Once the presence of hepatic steatosis is established, the “second hit”, oxidative stress, will further amplify the degree of steatosis [21]. OS has been proved to play an important role in hepatic cell damage and dysfunction [22] and could be induced by a high-fat diet. Furthermore, OS has been demonstrated to enhance insulin resistance and fat accumulation in the liver [23]. Recently, lipid peroxidation has been considered as the trigger factor responsible for the transition from simple fat accumulation to more progressive steatohepatitis or NASH [24]. Since hepatic steatosis might progress to NASH without timely therapy, and the seriousness of NAFLD is highly related to the degree of OS [25], it is urgent to improve antioxidative capacity to avoid the development of more serious hepatic pathologies.

Antioxidants have been suggested to slow the progression and attenuate NAFLD [7]. Dietary antioxidant components, including polyphenols and green tea extracts, have been verified to improve biochemical indexes and histological appearance in NAFLD [26,27]. Myricetin is a type of typical polyphenol widely distributed in edible plants with several therapeutic potential, including anti-diabetic, hypolipidemic, and hepatoprotective effects [12,28]. The purpose of this study was to explore the effects of myricetin on pre-existing hepatic steatosis induced by HFD, which would be most relevant to the clinical situation in humans. Previous research confirmed that high-fat diet (40.8% of calories from fat) consumption for nine weeks resulted in liver steatosis in C57BL/6 mice [29]. Here, we fed the same strain mice with a high-fat diet (45% of calories from fat) for 12 weeks and hepatic histological appearance confirmed that HFD induced hepatic steatosis model successfully. Myricetin treatment for 12 weeks caused a significant regression of pre-existing hepatic steatosis, as assessed by hepatic lipid concentration determinations and histological analysis. For specific performance, myricetin could remarkably reduce high-fat diet-induced alterations of hepatic TG, TC, and FFA contents, as well as lipid accumulation demonstrated by H & E staining and Oil Red O staining. Furthermore, myricetin was effective in reducing body weight and white adipose fat accumulation by increasing energy expenditure and dietary fat utilization suggested by significant lower RER values in indirect calorimetry analysis.

In order to have a deeper understanding of the possible mechanisms that might be responsible for the regressive effect of myricetin on hepatic steatosis, a genome-wide expression profiling in the liver tissues was performed and pathway analysis revealed that the pathways involved in hepatic lipid homeostasis, such as the PPAR signaling pathway, biosynthesis of unsaturated fatty acids, and the insulin signaling pathway, were all evidently affected. The PPAR signaling pathway that was most significantly affected was chosen to verify the expression of related DEGs, in which *Scd1*, *Lpl*, *Cd36*, and *Pparγ* were all significantly down-regulated, along with noticeable up-regulation of *Cyp7a1* by myricetin treatment compared with the HFD group. Previous studies demonstrated that the PPAR signaling pathway was involved in glucose homeostasis and lipid metabolism, and might be a target for the development of novel efficient treatments for several metabolic disorders, including obesity and type 2 diabetes [17]. PPARγ, a ligand-activated transcription factor which belongs to the nuclear receptor family, plays a vital role in lipid metabolism by regulating the expression of its target genes, such as *Scd1*, *Lpl*, and *Fasn* [30]. The genetic deletion of *Pparγ* in livers of either *ob/ob* [31] or AZIP-F-1 [32] mice significantly alleviated the development of hepatic steatosis, which was independent of the existence of hyperglycemia or hyperinsulinemia. Hepatic lipoprotein lipase (LPL), a target of PPARγ, exerts a vital role in lipoprotein metabolism. It was found that hepatic *Lpl* mRNA expression was higher in obese patients than normal controls [33] and reducing hepatic LPL activity was effective in ameliorating diet-induced obesity and hepatic steatosis [34]. *Cd36* is another target gene of PPARγ that could promote steatosis [35]. Results in the present study showed that myricetin treatment could decrease hepatic PPARγ protein expression, as well as normalizing the relative expression of its target genes, which might be a cause for its role in regression of hepatic steatosis.

The peroxisome pathway, which plays a critical role in redox signaling and lipid homeostasis, contributes to many crucial metabolic processes, such as fatty acid oxidation, biosynthesis of ether lipids, and free radical detoxification [36], was also changed by myricetin treatment. The involved DEGs, including *Sod2*, *Prdx1*, and *Prdx5*, which strongly connected with oxidative stress, were also up-regulated. We further measured the hepatic antioxidant enzymes, including SOD, CAT, and GPx, which were of fundamental importance in designing the therapeutic approaches toward oxidative-based liver pathologies [37], and found that these antioxidative enzymes were all significantly normalized by myricetin treatment. The TBARS levels were also remarkably decreased, declaring that myricetin improved hepatic steatosis possibly via alleviating oxidative stress.

Considering the fact that the Nrf2 pathway played a critical role in cytoprotection against oxidative stress through up-regulating phase II detoxifying enzymes [38], and that oxidative stress could be served as the “second hit” that activate simple steatosis to progress to NASH, we further explored whether myricetin alleviated hepatic steatosis via the Nrf2 pathway. Previous studies using mouse models have shown that activation of the Keap1/Nrf2 pathway, at least partially protected mice from diet-induced obesity and amelioration of hepatic steatosis [39,40]. As an effective antioxidant, myricetin has been reported to increase nuclear Nrf2 translocation [41] and ARE-binding activity to enhance Nrf2/ARE-mediated gene expressions [28]. Here we also showed that hepatic nuclear Nrf2 translocation was decreased by HFD and normalized by myricetin treatment. Moreover, hepatic NQO1 and HO-1 protein expression was also increased by myricetin, further indicating that myricetin reversed HFD-induced hepatic steatosis through the Nrf2 pathway, favoring enhancement of antioxidant capacity. However, studies have also indicated that the Nrf2 pathway activation attenuated inflammation-associated pathogenesis [42,43]. In the early phase of inflammation-mediated tissue damage, activation of Nrf2 signaling could inhibit the production or expression of pro-inflammatory mediators, including cytokines and chemokines [44]. Whether Nrf2 attenuated HFD-induced hepatic steatosis via inhibiting hepatic inflammation is unknown and still needs further research.

Nonetheless, there is evidence that PPARγ may directly regulate the expression of several antioxidant and prooxidant genes in response to OS, including *Cat*, *Sod2*, and *GPx* [45]. Emerging evidence also suggested that Nrf2 could crosstalk with metabolic pathways, increasing the repertoire of its target genes, such as *Pparγ* [46]. Meanwhile, PPARγ might act synergically with Nrf2 in the activation of antioxidant genes. Based on the present study, it is difficult to elucidate the underlying relationship between PPARγ and Nrf2 and further research is needed.

## 5. Conclusions

In conclusion, myricetin exhibited an excellent regressive effect against high-fat diet-induced hepatic steatosis, with such beneficial action accomplished via changing the PPAR signaling pathway and the Nrf2 pathway. These findings provide additional evidence in support of the use of myricetin as a promising functional food for the prevention or treatment of hepatic steatosis and other related metabolic disorders.

## Figures and Tables

**Table 1:** Regressive effects of myricetin on blood glucose, plasma insulin and lipid profiles.

	Con	CM	HFD	HM
Fasting blood glucose (mg/dL)	113.96 ± 11.51	117.24 ± 12.23	166.19 ± 15.79 *	123.35 ± 12.55 ^#^
Plasma insulin (μIU/mL)	14.47 ± 0.75	15.28 ± 1.93	20.63 ± 2.06 *	14.94 ± 1.46 ^#^
HOMA-IR	4.05 ± 0.45	4.47 ± 0.87	8.67 ± 1.39 *	4.61 ± 0.60 ^#^
Plasma TG (mmol/L)	2.53 ± 0.15	2.54 ± 0.16	3.36 ± 0.17 *	2.71 ± 0.15 ^#^
Plasma TC (mmol/L)	4.09 ± 0.24	4.01 ± 0.27	6.57 ± 0.37 *	4.35 ± 0.38 ^#^
Plasma HDL-C (mmol/L)	1.81 ± 0.08	1.92 ± 0.08	1.41 ± 0.05 *	1.69 ± 0.08 ^#^
Plasma LDL-C (mmol/L)	2.08 ± 0.12	2.05 ± 0.09	2.81 ± 0.10 *	2.30 ± 0.07 ^#^

Values were expressed as mean ± SEM (*n* = 8). Con, control diet; CM, control diet with 0.12% additional myricetin; HFD, high-fat diet; HM, high-fat diet with 0.12% additional myricetin; HOMA-IR, homeostatic model assessment index of insulin resistance; TG, triglyceride; TC, total cholesterol; HDL-C, high-density lipoprotein cholesterol; LDL-C, low-density lipoprotein cholesterol. * *p* < 0.05 vs. the Con group; ^#^ *p* < 0.05 vs. the HFD group.

**Table 2:** Significantly altered biological pathways in livers of the HM mice compared to HFD mice.

Number	Pathway Name	Input Genes in Pathway	Impact Factor	Corrected Gamma *p*-Value	Significantly Altered Genes
1	PPAR signaling pathway	6	16.26	1.90 × 10^−5^	*Cd36*, *Scd1*, *Cyp7a1*, *Lpl*, *Pparg*, *Pck1*
2	Biosynthesis of unsaturated fatty acids	3	10.26	0.00185	*Acot1*, *Acot3*, *Scd1*
3	Peroxisome	3	10.01	0.001974	*Sod2*, *Prdx5*, *Prdx1*
4	Complement and coagulation cascades	3	9.62	0.002971	*C4b*, *Plg*, *Serpina1a*
5	Renal cell carcinoma	3	8.26	0.008088	*Arnt*, *Jun*, *Raf1*
6	Long-term potentiation	4	7.02	0.01977	*Araf*, *pla2g2d*, *Plcb1*, *Raf1*
7	GnRH signaling pathway	3	6.44	0.029597	*Jun*, *Pla2g6*, *Raf1*
8	Toll-like receptor signaling pathway	3	5.92	0.032924	*Cxcl10*, *Jun*, *Spp1*
9	Insulin signaling pathway	3	5.64	0.041843	*Pck1*, *Raf1*, *Srebf1*
10	MAPK signaling pathway	3	5.60	0.043097	*Jun*, *Ntrk2*, *Pla2g6*
